# Coordinated Biosynthesis of Essential Cell Envelope Components: Lipopolysaccharide and Fatty Acids Requires LapD, Acyl Carrier Protein, and Fully Hexaacylated Lipid A

**DOI:** 10.3390/ijms262210993

**Published:** 2025-11-13

**Authors:** Marta Jeschke, Aravind Ayyolath, Akshay Maniyeri, Satish Raina, Gracjana Klein

**Affiliations:** Laboratory of Bacteria Genetics, Gdansk University of Technology, 80-233 Gdansk, Poland

**Keywords:** lipopolysaccharide (LPS), fatty acid biosynthesis FASII, myristoyltransferase, heptosyltransferase, acetyl-CoA carboxylase, LapD, LapB, phospholipids

## Abstract

Lipopolysaccharide (LPS) is an essential component of the outer membrane (OM) of Gram-negative bacteria, and its levels are tightly co-regulated with phospholipid (PL) amounts. This homeostatic regulation necessitates the involvement of numerous genes, including *lapD* in a poorly defined manner. To understand the function of LapD, we took advantage of the synthetic lethal phenotype conferred by the concomitant absence of LapD and myristoyltransferase LpxM or heptosyltransferase WaaC and isolated extragenic suppressors that could bypass this lethality. Suppressor analyses of Δ(*lapD lpxM*) bacteria identified five single amino acid exchanges in AccA and two in each of AccC and AccD. These proteins comprise different subunits of the acetyl-CoA carboxylase complex, which catalyzes the rate-limiting step in the initiation of fatty acid synthesis, mediating the conversion of acetyl-CoA to malonyl-CoA. Fatty acid analysis revealed that these mutations restored the ratio of saturated to unsaturated fatty acids and repressed elevated PL levels. Suppressor analyses of Δ(*lapD waaC*) identified a single amino acid substitution in LptD, which is required for LPS assembly in the OM, and in NlpI, which regulates the amount of peptidoglycan hydrolase MepS. These results posit LapD as the point of critical regulation of homeostatic control of three essential cell envelope components.

## 1. Introduction

The cell envelope of Gram-negative bacteria, including *Escherichia coli*, contains two distinct membranes, an inner (IM) and an outer (OM) membrane, separated by the periplasm, a hydrophilic compartment that includes a layer of peptidoglycan (PGN). The OM is essential for the viability of Gram-negative bacteria. This OM is asymmetric in nature due to the location of lipopolysaccharide (LPS) in its outer leaflet and phospholipids (PLs) directed inward [1]. The maintenance of this asymmetry is crucial for the integrity of the bacterial cell envelope. LPS is the major component of OM, covering nearly 70% of OM, and is the key virulence factor and causative agent of sepsis caused by Gram-negative bacteria. LPSs are highly heterogeneous in composition [2]. However, they share a common architecture composed of a membrane-anchored phosphorylated and acylated *β*(1⟶6)-linked GlcN disaccharide, termed lipid A, to which a carbohydrate moiety of varying size is attached [3]. In *E*. *coli*, the precursor lipid IV_A_ acts as an acceptor for the transfer of two 3-deoxy-α-D-*manno*-oct-2-ulosonic acid (Kdo) residues by WaaA, generating the key Kdo_2_-lipid IV_A_ intermediate, which serves as a substrate for the transfer of two additional fatty acids that require LpxL and LpxM enzymes [4,5]. All four lipid A acyltransferases, LpxA, LpxD, LpxL, and LpxM, are dependent on acyl chain activation by the acyl carrier protein AcpP, as coenzyme A thioesters do not serve as substrates [4,6]. AcpP also participates in several steps of fatty acid biosynthesis. Thus, a balanced cell envelope composition involves inbuilt mechanisms to sustain a balance between the LPS and glycerophospholipids biosynthetic pathways, as excess of either component causes lethality.

**Figure 1:**
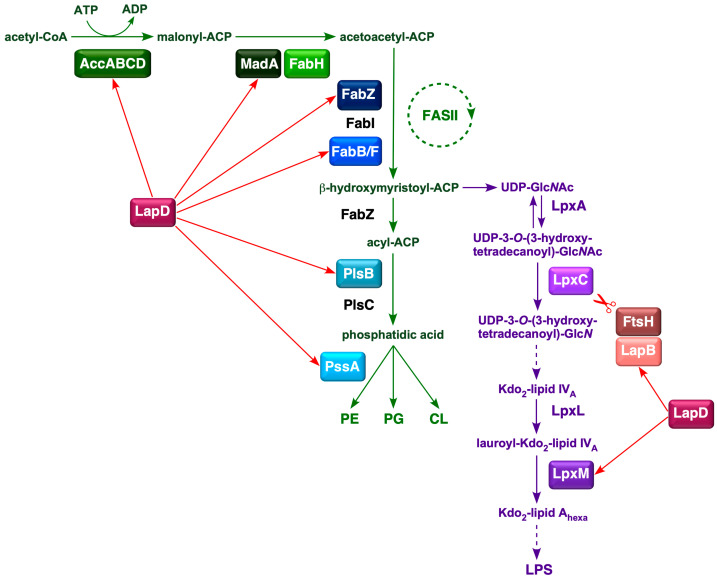
Schematic drawing of LapD-mediated coupled PL and LPS biosynthesis in *E*. *coli*. Various steps in fatty acid biosynthesis, with initiation catalyzed by the acetyl-CoA carboxylase enzyme and entry into the FASII cycle, are shown. The intermediate *β*-hydroxymyristoyl-ACP serves as a precursor in the subsequent steps of PL and LPS biosynthesis. Red arrows depict the role of LapD based on its co-purification with specific proteins involved in fatty acid and LPS biosynthesis, and genetic interactions based on the synthetic lethality with Δ*lapD* in mutational combinations.

Bacteria maintain a tight balance between the amounts of LPS and PL because they use (*R*)-3-hydroxymyristate-ACP as a common metabolic precursor [7]. In *E*. *coli*, this balance is achieved by regulating the amount of LpxC, which catalyzes the first committed step in LPS biosynthesis [8]. The LpxC enzyme is unstable, and its amount is determined by FtsH-mediated proteolysis and several poorly defined signals, such as bacterial growth rate, ppGpp levels, fatty acid composition, FabZ, FabI, FabK activities, and the accumulation of lipid A precursors [8,9,10,11,12]. However, FtsH requires another essential protein, LapB, to catalyze LpxC proteolysis [11]. The proteolysis of LpxC is counteracted by LapC, which is thought to be adjusted depending on the accumulation and demand for LPS synthesis [13,14,15,16,17,18,19]. LapC and LapB interact genetically and physically [13,15]. Current models suggest that LapC senses the accumulation of LPS in the IM and can bind to either LPS or LapB [7,19]. Binding of LapC to LPS, instead of LapB, promotes LapB–FtsH interaction to degrade LpxC to overcome the lethal accumulation of LPS in the IM. However, the mechanism by which bacteria couple LPS and phospholipid synthesis remains unclear. In this process, LapD, another protein that interacts with LapB, could be a potential link in coupled fatty acid and LPS biosynthesis, as it forms a complex with several enzymes involved in fatty acid and phospholipid biosynthesis (Figure 1) [20].

The fatty acid biosynthesis pathway in *E. coli* is a type II FAS system [21]. During this process, acyl intermediates are covalently conjugated to ACP via a thioester linkage. *E. coli* synthesizes four major fatty acids, C_14:0_(myristate), C_16:0_(palmitate), C_16:1_(palmitoleate), and C_18:1_(*cis*-vaccenate), and two minor components, laurate (C_12:0_) and 3-hydroxymyristate. The latter two are components of lipid A, whereas palmitate, palmitoleate, and *cis*-vaccenate are phospholipid components. Fatty acids form the acyl chains found in all PLs. *E. coli* can also take up exogenous fatty acids and convert them into acyl-CoA. Acyl-CoA molecules constitute a separate pool from endogenously synthesized acyl-ACPs [22]. Acyl-CoA can be used for phospholipid synthesis or broken down by *β*-oxidation. Fatty acid synthesis is also the primary biosynthetic determinant of *E. coli* size [23]. Regulation of the first step in PL synthesis, catalyzed by the PlsB enzyme, which synthesizes lysophosphatidic acid from long-chain ACP and *sn*-glycerol-3 phosphate, is another point of coupling between PL and LPS biosynthesis [24] (Figure 1).

Currently, the precise function of LapD remains unknown. One study implicated LapD in cell division [25]. Another study suggested that LapD plays a role in diverse cellular envelope-related functions, including PGN biosynthesis [26]. However, no direct proof has been provided concerning the involvement of LapD in these processes. Prior to the above-mentioned studies, our laboratory has shown that LapD is required for growth at critical high temperatures [27]. Subsequently, we presented evidence suggesting a role in LPS and fatty acid biosynthesis [20] (Figure 1). First, purification of LapD revealed that it co-purifies with several enzymes that participate in the initiation of fatty acid biosynthesis, AccD, MadA (FabY), and those mediating committed steps of FASII (FabH, FabF, and FabB) [20]. Second, the temperature sensitivity (Ts) of Δ*lapD* bacteria can be effectively suppressed by overexpression of the *acpP* gene or by an imbalance in the synthesis of acetyl coenzyme A (acetyl-CoA) carboxylase (ACC) complex (due to overexpression of one of the subunits of the ACC complex) [20]. ACC enzyme catalyzes the first committed step in fatty acid biosynthesis, which is the conversion of acetyl-CoA to malonyl-CoA [28]. Curiously, overexpression of the *acpP* gene or one of the genes encoding ACC subunits suppresses Δ*lapD* phenotypic defects without increasing LpxC levels, suggesting an alternative role in linking fatty acid and LPS coordination [20]. In support of our findings, it has been suggested that the absence of LapD causes the activation of fatty acid biosynthesis [29]. As Δ*lapD* bacteria have reduced LPS levels, an increase in LPS amounts by the introduction of either stable variants of LpxC or inactivation of the *lapB* gene can relieve some of the phenotypic defects of Δ*lapD* bacteria [20].

Here, we used multipronged approaches to unravel the physiological function of LapD. LapD by itself is non-essential for bacterial viability, except at temperatures above 44.5 °C or when the growth medium is supplemented with vancomycin [20,26]. However, the lack of LapD causes a reduction in LpxC at elevated temperatures and reduces the amount of LPS in the OM due to the retention of significant amounts in the IM [20]. Interestingly, we recently showed that LapD becomes essential in the absence of myristoyltransferase LpxM, and Δ*lapD* bacteria also exhibit synthetic growth defects in the absence of heptosyltransferase WaaC [20]. These phenotypes were used to isolate extragenic suppressors that bypass this lethality. Single-copy chromosomal suppressors of Δ(*lapD lpxM*) mapped mostly to genes encoding different subunits of the acetyl-CoA carboxylase enzyme, which mediates the first step in fatty acid biosynthesis. These suppressors significantly altered fatty acid levels and reduced elevated PL levels. Similarly, suppressors of Δ(*lapD waaC*) synthetic lethality were isolated and shown to map to the *lptD* gene, whose product is required for LPS assembly in the OM, and to the *nlpI* gene. NlpI regulates the amount of peptidoglycan hydrolases, such as MepS, by acting as an adaptor for Prc protease. We further show that overexpression of *acpP* and *accB* genes overcomes cell morphology defects of Δ*lapD* bacteria and a reduction in PL amounts. These results allow us to conclude that LapD in concert with LpxM is required to regulate balanced amounts of fatty acids and to couple LPS, PL, and PGN synthesis.

## 2. Results

### 2.1. Suppressor-Free Δ(lapD lpxM) Bacteria Are Viable in Minimal Medium Under Slow Growth Conditions but Require Extragenic Suppressors for the Growth in Rich Medium

**Figure 2:**
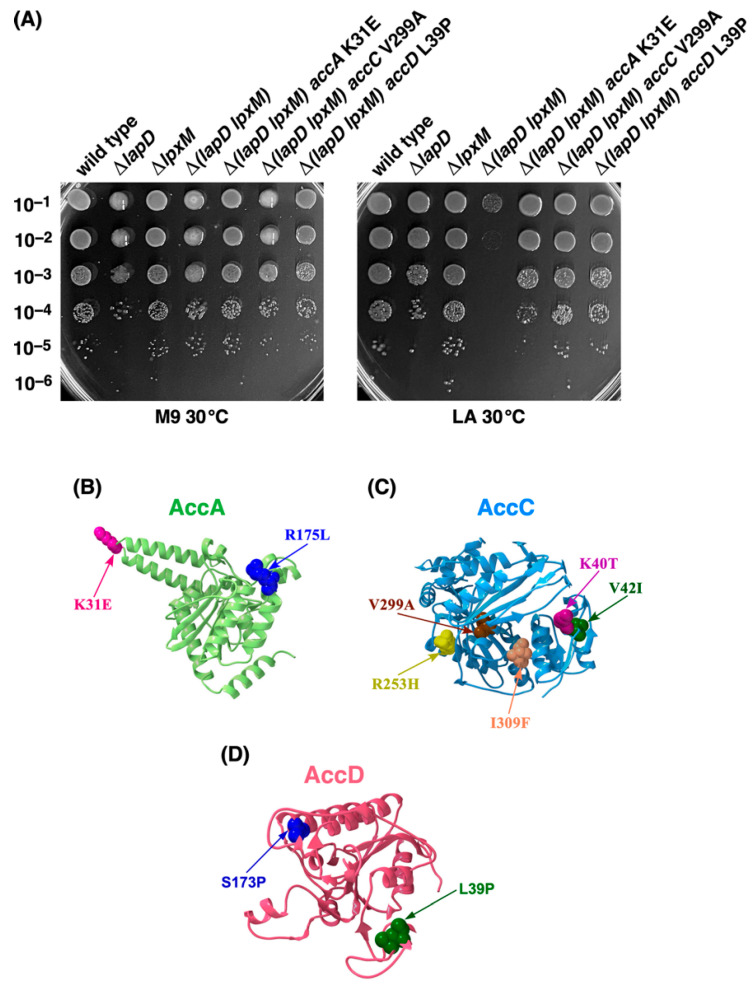
Mutations mapping to *accA*, *accC*, and *accD* genes encoding various subunits of the acetyl-CoA carboxylase enzyme overcome the synthetic lethality of Δ(*lapD lpxM*) bacteria on LA medium. (**A**) Growth of isogenic cultures of the wild type, its Δ*lapD*, Δ*lpxM*, and Δ(*lapD lpxM*) derivatives with and without suppressor mutations mapping to various *acc* genes was quantified by spot dilution on M9 minimal and LA media. The relevant genotypes and incubation temperature are indicated. (**B**) Positions of various single amino acid substitutions in the structure of AccA PDB 8UZ2, (**C**) AccC PDB 1BNC and (**D**) AccD PDB 2F9Y are shown.

We previously showed that the Δ(*lapD lpxM*) mutational combination is lethal and Δ(*lapD waaC*) bacteria exhibit conditional lethality [20]. To understand the molecular basis of this lethality, we initiated several approaches based on the isolation and characterization of extragenic chromosomal suppressors that overcome this synthetic lethality in the W3110 background, which is wild type for the *fabR* gene, unlike BW25113, which has a non-functional *fabR* gene [30]. Viable suppressor-free Δ(*lapD lpxM*) transductants could not be obtained on LA medium at either 30 or 37 °C unless a wild-type copy of either the *lapD* gene or the *lpxM* gene was provided from a plasmid (Table 1). Because strains synthesizing LPS composed of only lipid IV_A_ are viable on M9 minimal medium at low temperatures [31], we used the same approach to construct Δ(*lapD lpxM*) derivatives in the wild-type W3110 background. Δ(*lapD lpxM*) transductants could be obtained readily on the minimal medium at 30 °C at the same frequency, when only an empty vector was present in the recipient or with the ectopic presence of either *lapD* or *lpxM* on the plasmid (Table 1). However, when Δ(*lapD lpxM*) derivatives grown in M9 medium were analyzed for bacterial growth on LB medium at either 30 or 37 °C, they exhibited severe growth defects and were unable to form colonies (Figure 2). These results imply that under slow growth conditions of minimal medium, viable suppressor-free Δ(*lapD lpxM*) can be constructed at 30 °C; however, they cannot be propagated in rich medium even at 30 °C. The construction and viability of suppressor-free Δ(*lapD lpxM*) on minimal medium at 30 °C provided an excellent tool to study their properties and isolate suppressors that overcome their synthetic lethality on rich medium.

### 2.2. Suppressor Mutations Mapping to Genes Encoding Different Subunits of Acetyl Coenzyme A Carboxylase Can Bypass the Lethal Phenotype of Δ(lapD lpxM) Bacteria

To address the reasons for the lethality of Δ(*lapD lpxM*) bacteria under fast-growing conditions, suppressor-free, independently isolated transductants of such bacteria were grown in M9 minimal medium (permissive growth conditions) and plated on LA medium at 37 and 42 °C. Marking and mapping of suppressor mutations with Tn*10* and further transduction of individual suppressor mutations allowed the growth of the suppressor-free Δ(*lapD lpxM*) GK6173 strain on LA medium at 30, 37, and 42 °C in 16 out of 23 strains, and thus were not strain-specific suppressor mutations constituting six complementation groups with three covering genes encoding subunits of the ACC complex. DNA sequence analysis of PCR products from one complementation group revealed that they have a single amino acid exchange in the *accA* gene (K31E, R175L) (Table 2). In the second complementation group, which included six strains, DNA sequence analysis revealed five single amino acid suppressor mutations in the *accC* gene (K40T, V42I, R253H, V299A, and I309F). A similar strategy revealed that the third complementation group contained single amino acid substitutions in the *accD* gene (L39P and S173P). The amino acid exchanges of V299A in *accC*, and K31E and R175L in *accA* genes were recovered in two independently isolated Ts^+^ derivatives of Δ(*lapD lpxM*) (Table 2).

The ability to restore the growth of Δ(*lapD lpxM*) bacterial strains in the presence of various extragenic suppressor mutations mapping to the ACC complex was quantified using spot-dilution experiments. These analyses revealed that suppressor mutations mapping to either *accA*, *accC*, or *accD* in the Δ(*lapD lpxM*) background restored colony-forming ability on LA medium to nearly wild-type levels (Figure 2). These conditions are lethal to the parental Δ(*lapD lpxM*) strain, which, without suppressors, can grow only on M9 minimal medium at 30 °C (Figure 2). Genomic DNA of the remaining seven Ts^+^ suppressor strains was subjected to whole-genome sequencing but could not be followed because of the presence of more than one mutation.

In *E*. *coli*, the acetyl-CoA carboxylase complex is heteromultimeric and comprises multiple subunits of AccA, AccB, AccC, and AccD. In the initiation of fatty acid biosynthesis, the major regulation is exerted at the level of the acetyl-CoA carboxylase complex, as it constitutes a rate-limiting step and is required to produce malonyl-CoA by the carboxylation of acetyl-CoA [28]. The ACC complex is subjected to feedback inhibition because its activity is inhibited by acylated derivatives of AcpP [32,33]. Hence, the loss-of-function mutations that we obtained as suppressors of Δ(*lapD lpxM*) bacteria are expected to cause an overall reduction in the biosynthesis of fatty acids/PL.

### 2.3. Suppressor Mutations in Acetyl-CoA Carboxylase Complex That Bypass the Lethal Phenotype of Δ(lapD lpxM) Bacteria Accumulate Reduced Amounts of Phospholipid Species

**Figure 3:**
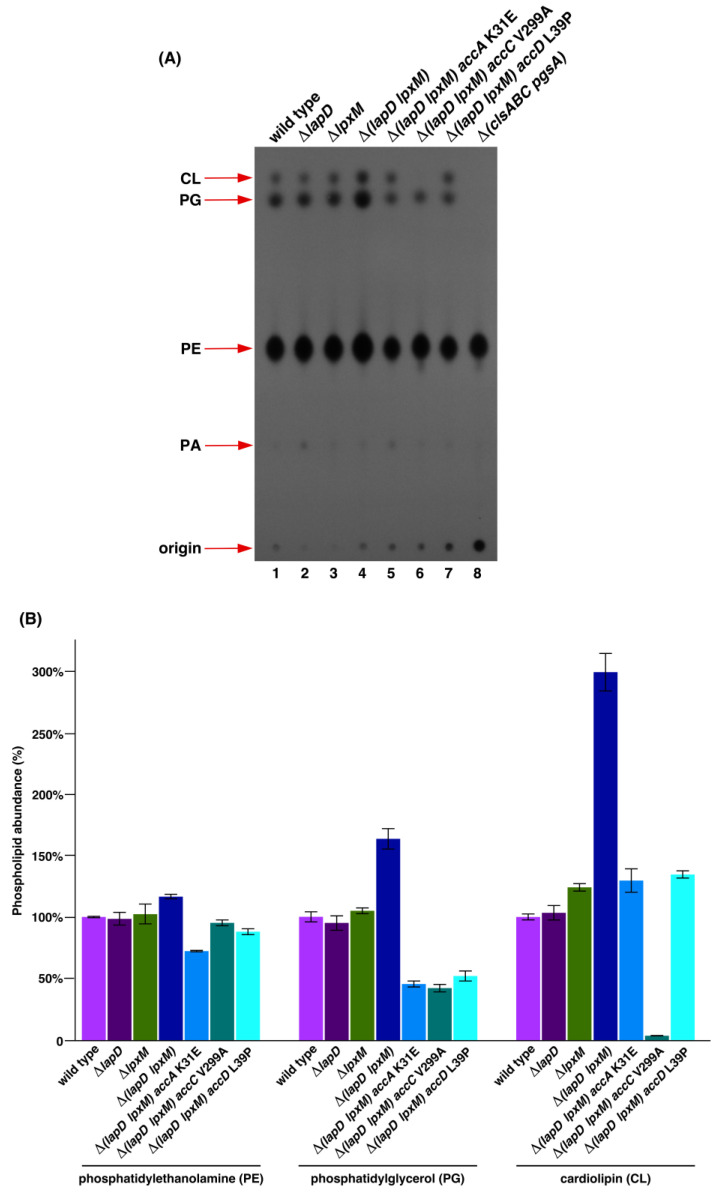
Δ(*lapD lpxM*) bacteria have excess of phospholipids and suppressor mutations in genes encoding various subunits of acetyl-CoA carboxylase enzyme that overcome their synthetic lethality act by decreasing phospholipid synthesis. (**A**) Thin-layer chromatography of ^32^P-labeled PLs extracted from isogenic strains derived from Δ(*lapD lpxM*) bacteria carrying suppressor mutations in *accA*, *accC*, and *accD* genes. In parallel, ^32^P-labeled PLs were extracted from the parental wild-type, Δ*lapD*, and Δ*lpxM* bacteria, with samples from the RU857 strain serving as a marker for the migration of PL species. A representative image of from three biological replicates is shown. The arrows indicate the positions of major PL species. (**B**) Bar graph representing the relative amounts of CL, PG, and PE species quantified using densitometry and quantification by phosphorimaging analysis from triplicate biological repeats are presented. The S.E and relevant genotype are indicated.

Fatty acid and phospholipid biosynthesis are intricately linked. Thus, we analyzed the PL pattern of isogenic parental wild-type W3110 and its Δ*lapD*, Δ*lpxM*, Δ(*lapD lpxM*), and Δ(*lapD lpxM*) derivatives with chromosomal single amino acid substitutions in *accA*, *accC*, and *accD* genes using thin-layer chromatography (TLC). As an additional control and to serve as a marker for PL species, we labeled cultures of strain RU857, which cannot synthesize cardiolipin (CL) and phosphatidylglycerol (PG) species. PL species were visualized by phosphorimaging analysis and densitometry. These experiments did not show any significant differences in the total PL amounts of the three PL species between isogenic wild-type and Δ*lapD* bacteria. However, densitometric analysis of the PL species of Δ*lpxM* bacteria revealed an approximately 24% increase in the abundance of CL species. Most significantly, there was an overall higher accumulation of PL species in Δ(*lapD lpxM*) bacteria, with a nearly 64% increase in PG and approximately 3-fold elevated levels of CL species as compared to those in Δ*lapD* or wild-type bacteria (Figure 3 lane 4). Significantly, all three suppressor-containing strains of Δ(*lapD lpxM*) with mutations in the ACC complex exhibited a remarkable reduction in the incorporation of ^32^Pi in PE, PG, and CL species compared to the parental Δ(*lapD lpxM*) strain (Figure 3 lanes 5 to 7). This reduction in the accumulation of PLs in ACC suppressor-containing strains is particularly evident for PG and CL species, which are otherwise elevated in Δ(*lapD lpxM*) bacteria. The most noticeable results were from the strain SR25219 Δ(*lapD lpxM*) with *accC* V299A exchange, which showed a reduction in PG amounts of more than 60% compared to the parental strains, with only trace amounts of CL species (only 3% of the wild-type level) (Figure 3 lane 6) and nearly unaffected levels of PE. Interestingly, SR25218 Δ(*lapD lpxM*) *accA* K31E exhibited a nearly 30% reduction in PE and approximately 55% reduction in PG levels. Examination of PL amounts in SR25217 Δ(*lapD lpxM*) *accD* L39P also revealed a nearly 48% decrease in PG levels (Figure 3). Thus, these suppressor mutations in the ACC complex that relieve toxicity associated with Δ(*lapD lpxM*) act by reducing PL synthesis.

### 2.4. Suppressor Mutations in Acetyl-CoA Carboxylase Complex That Bypass the Lethal Phenotype of Δ(lapD lpxM) Bacteria Are Recessive

**Figure 4:**
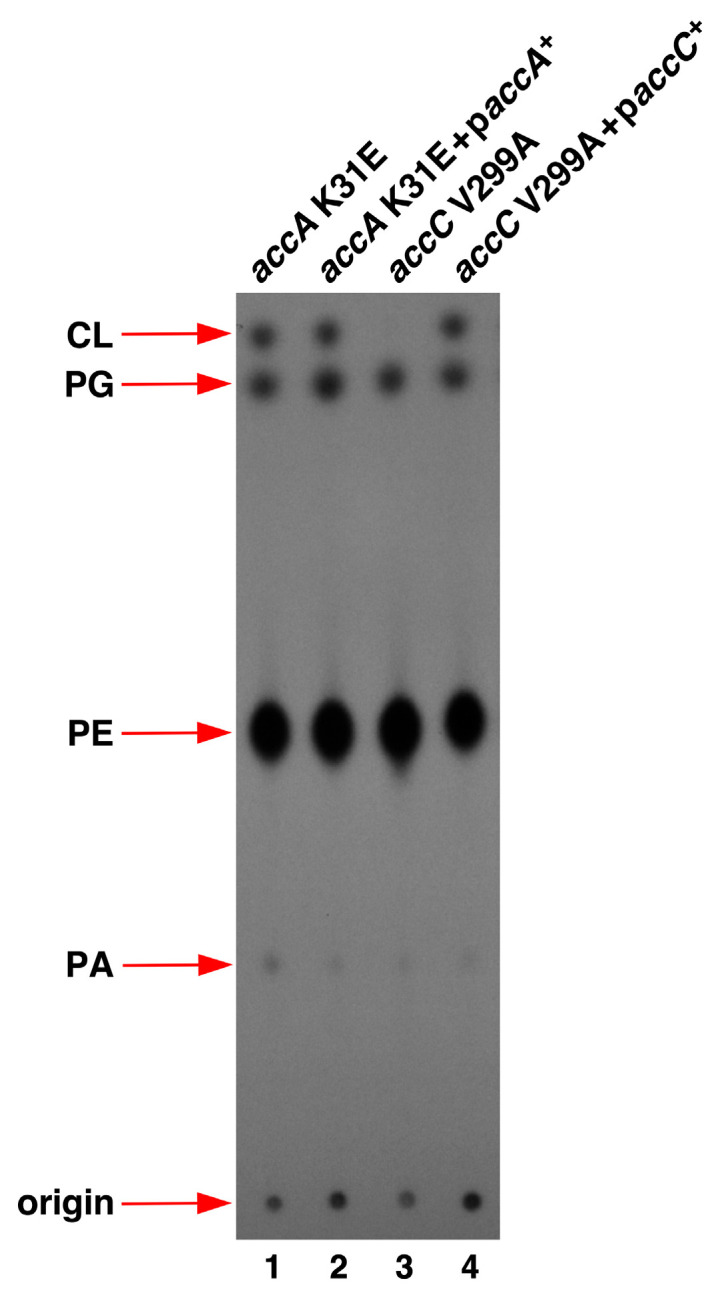
Suppressor mutations in *accA* and *accC* genes that overcome the lethal phenotype of Δ(*lapD lpxM)* bacteria are recessive. Thin-layer chromatography of ^32^P-labeled PLs extracted from isogenic strains derived from Δ(*lapD lpxM*) bacteria carrying suppressor mutations in *accA* and *accC* genes, and their isogenic derivatives transformed with single-copy complementing cosmid clones. A photographic image of a representative autoradiogram from three biological replicates is shown. The arrows indicate the positions of major PL species.

It became imperative to verify whether the suppressor mutations identified in *acc* genes were loss-of-function mutations. To address this, mutations in *accA*, *accC*, and *accD* were transduced using the linked Tn*10* Cm marker. The same cosmid clones (single-copy) that were used to recombine the Tn*10* marker were used for complementation and analyzed for PL content using TLC. This analysis revealed restoration of the abundance of PG, and CL species, which were diminished in *accA* and *accC* mutants using complementing cosmid clones for *accA* K31E and *accC* V299A (Figure 4, lanes 1 vs. 2 and lanes 3 vs. 4).

These data allow us to conclude that mutations in genes encoding the ACC complex are recessive, which lead to the reduction in PL species.

### 2.5. Suppressor Mutations in Acetyl-CoA Carboxylase Complex That Overcome the Lethal Phenotype of Δ(lapD lpxM) Bacteria Do Not Alter LpxC Levels

**Figure 5:**
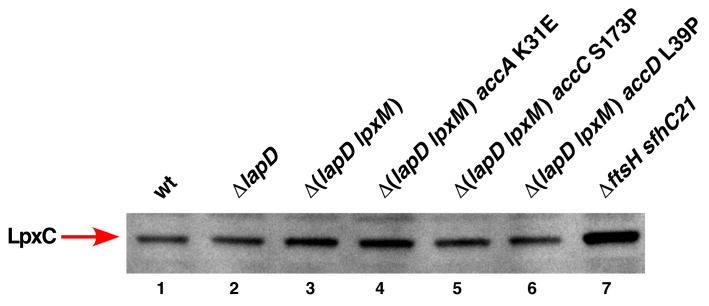
LpxC levels are unaltered by suppression mutations in *accA* and *accC* genes. Immunoblot of whole-cell lysates obtained from isogenic strains grown at 30 °C in M9 minimal medium. An equivalent amount of total protein was resolved by 12% SDS-PAGE prior to immunoblotting. For immunoblotting, LpxC-specific antibodies were used, and the relevant genotype is indicated. As an internal control, a cell lysate from the isogenic Δ*ftsH sfhC21* strain was applied (lane 7).

To rule out that suppressor mutations mapping to genes encoding the ACC complex do not alter LpxC amounts, immunoblot analysis was performed. Isogenic cultures of wild type, Δ*lapD*, Δ(*lapD lpxM*), and its derivatives with suppressor mutations in *accA*, *accC*, and *accD* genes were grown under permissive growth conditions. Equivalent amounts of proteins were transferred by Western blotting and immunoblotted with anti-LpxC antibodies. Estimation of LpxC levels revealed that they were nearly unaltered by the presence of suppressor mutations in *acc* genes (Figure 5). As a control for LpxC levels, equivalent amounts of proteins from the cell lysate of Δ*ftsH sfhC21* bacteria were applied, which had expectedly elevated amounts of LpxC due to its stabilization (Figure 5 lane 7).

### 2.6. Lethality of Δ(lapD lpxM) Bacteria Due to Gross Changes in the Amounts of Free Fatty Acids That Can Be Bypassed by Mutations in the ACC Complex

As shown in the above sections, the concomitant absence of LapD and myristoyl acyltransferase LpxM causes significant changes in the phospholipid content. Thus, it became imperative to examine the fatty acid composition of Δ(*lapD lpxM*) bacteria and their isogenic suppressors that overcome the lethal phenotype. Thus, free fatty acids (FFAs) were extracted in hexane and examined using gas chromatography. Two sets of growth conditions were used, with one condition as described in the PL compositional analysis, with a brief shift to LB medium, and another when bacterial cultures were grown only in minimal medium, since in these experiments, bacteria were not labeled with ^32^Pi. These experiments revealed that Δ(*lapD lpxM*) bacteria have nearly a 2-fold increase in myristic acid (C14:0), and the levels are restored by suppressor mutations in *acc* genes under M9 minimal medium growth conditions. This is best exemplified by the restoration of wild-type-like amounts of myristic acid, particularly by the *accC* V299A mutation. Under the same growth conditions, the amounts of unsaturated fatty acids (UFAs) C16:1 palmitoleic acid increased by approximately 2-fold in Δ(*lapD lpxM*) bacteria. This phenotype is repressed by suppressor mutations in either *accA* or *accC* genes. This analysis further showed that the amounts of *cis*-vaccenic acid C18:1 were reduced in Δ(*lapD lpxM*) bacteria by approximately 22% compared to those measured in the isogenic wild-type strain when grown at 30 °C, but not at 37 °C. Interestingly, suppressor mutation mapping to the *accC* gene caused a significant increase in the amount of *cis*-vaccenic acid (more than 1.6-fold) compared to that in the wild type and a 2-fold increase over that measured in Δ(*lapD lpxM*) bacteria at 30 °C. Consistent with these results, even when FFAs were extracted from bacteria grown at 37 °C, the amount of *cis*-vaccenic acid significantly increased (Table 3). These results suggest that the absence of myristoyl acyltransferase in Δ*lapD* bacteria results in large-scale changes in the biosynthesis of fatty acids, some of which are reversed by suppressor mutations in genes encoding different subunits of the ACC complex.

### 2.7. Inhibition of Fatty Acid Biosynthesis by Exogenous Addition of Either Triclosan or Cerulenin Overcomes Lethality of Δ(lapD lpxM) Bacteria

**Figure 6:**
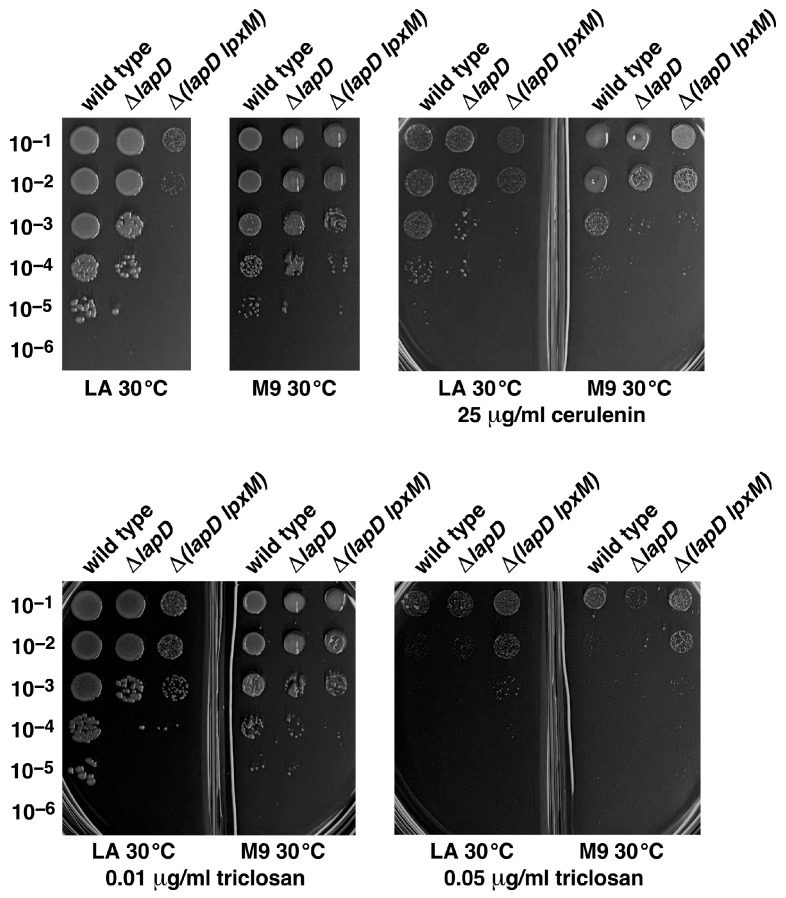
Inhibition of fatty acid biosynthesis by the addition of either cerulenin or triclosan can overcome the lethal phenotype of Δ(*lapD lpxM*) bacteria. Growth of isogenic cultures of wild type, its Δ*lapD*, and Δ(*lapD lpxM*) derivatives was quantified by spot dilution on M9 minimal medium and LA alone or when supplemented with either 25 µg/mL of cerulenin (upper panel) or with 0.01 or 0.05 µg/mL of triclosan. The relevant genotypes and incubation temperature are indicated. Split plates with either M9 or LA were used for spot dilution. The concentrations of cerulenin or triclosan and relevant genotypes are indicated.

As Δ(*lapD lpxM*) bacteria exhibit an increase in the abundance of PL species, indicating excessive fatty acid synthesis contributing to lethality, we hypothesized that the inhibition of fatty acid synthesis could overcome the synthetic lethal phenotype. Thus, we supplemented the growth medium with sublethal doses of inhibitors, such as triclosan and cerulenin. Triclosan is a well-characterized inhibitor of FabI enzyme, the enoyl-ACP (acyl-carrier protein) reductase component of FASII [34]. Cerulenin inhibits the elongation steps of fatty acid biosynthesis by irreversibly binding to FabB and FabF enzymes [35]. After titration experiments, we supplemented M9 minimal and LA growth medium with 0.01 and 0.05 µg/mL of triclosan and 25 µg/mL of cerulenin, the concentration tolerated by the wild-type bacteria, although the colony size was reduced and viability was reduced by a factor of 10-fold (Figure 6). Using spot dilution assays, we found that supplementation of LA medium with either triclosan or cerulenin slightly promoted the growth of Δ(*lapD lpxM*) bacteria at 30 °C (Figure 6). As shown earlier and in the control experiments, Δ(*lapD lpxM*) bacteria did not grow on LA medium. Interestingly, on M9 and LA media, supplementation with 0.05 µg/mL of triclosan was tolerated by Δ(*lapD lpxM*) bacteria but not by isogenic Δ*lapD* bacteria, indicating that excess fatty acid biosynthesis is one of the reasons for the lethal phenotype of Δ(*lapD lpxM*) bacteria.

### 2.8. Suppressor Mutations Mapping to opgG, aceK and mukE Genes Can Bypass the Lethal Phenotype of Δ(lapD lpxM) Bacteria at 37 °C

**Figure 7:**
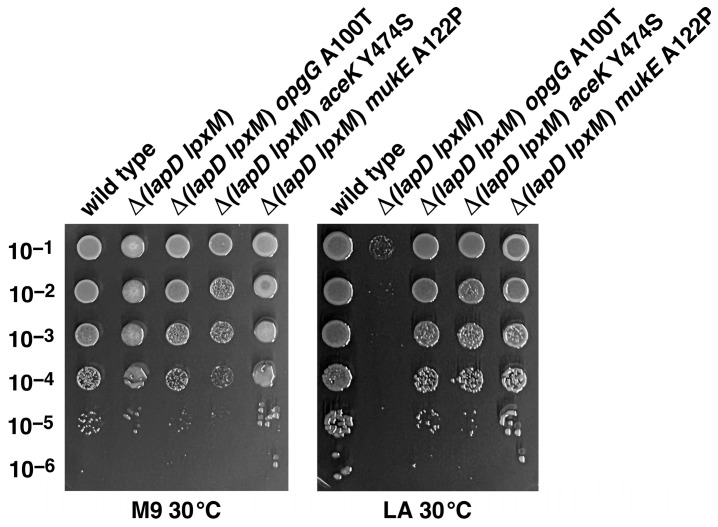
Single amino acid substitutions in *opgG*, *aceK* and the *mukE* gene can overcome the synthetic lethality of Δ(*lapD lpxM*) bacteria. Growth of isogenic cultures of wild type, Δ(*lapD lpxM)*, its isogenic derivatives with various suppressor mutations was quantified by spot dilution on M9 and LA 30 °C. Plates were incubated for 24 h. Data from a representative experiment with indicated genotype are shown.

As mentioned above, Tn*10*-linked suppressor mutation of Δ(*lapD lpxM*) bacteria fell into six complementation groups. Using the same strategy after marking with Tn*10*, mapping of three remaining sets and DNA sequence of relevant PCR products revealed that two strains, SR25437 and SR25443, contained a single amino acid exchange of A100T in the *opgG* gene (Table 2). DNA sequencing of PCR products of relevant genomic regions identified two additional Δ(*lapD lpxM*) suppressor-carrying strains, SR25452 and SRSR25458, with single amino acid exchanges in *aceK* (Y474S) and *mukE* (A122P) genes, respectively. The restoration of growth of Δ(*lapD lpxM*) bacteria by suppressor mutations mapping to *opgG*, *mukE*, and *aceK* was quantified by growth measurement using spot-dilution assays (Figure 7). The *aceK* gene encodes isocitrate dehydrogenase kinase/phosphatase (AceK), which controls the branch point between two pathways of central metabolism: the TCA cycle and the glyoxylate cycle [36]. Mutation in the *aceK* gene could potentially alter the levels of precursor acetyl-CoA used in these cycles and also feeds into fatty acid biosynthesis. Significantly, amino acid residue Y474 is located next to the catalytically essential amino acid residue D475, whose inactivation abolishes the kinase activity of AceK [37]. The active site of AceK contains a highly conserved VVF**Y**DYDEI motif (Y474 is in bold) [38]. OpgG is a periplasmic protein required for the synthesis of branched periplasmic oligosaccharides, which requires UDP-glucose and the AcpP [39,40].

MukE is involved in chromosome partitioning, and its loss affects the focal localization of the MukBEF complex [41,42,43]. MukB, MukE, and MukF are also interacting partners of LapD [20]. Taken together, suppressors mapping to either the ACC complex or in *aceK* or *mukE* genes could regulate fatty acid amounts, the amount of acyl-CoA precursor and cell division, providing a reasonable explanation for their isolation and the molecular basis of the synthetic lethal phenotype of Δ(*lapD lpxM*) bacteria.

### 2.9. Suppressor Mutations Mapping to lptD and nlpI Genes Can Bypass the Synthetic Lethal Phenotype of Δ(lapD waaC) Bacteria at 42 °C

We previously showed that Δ*lapD* bacteria exhibit a synthetic lethal phenotype in the absence of WaaC heptosyltransferase at temperatures above 42 °C, which are otherwise permissive for deletions in either *lapD* or *waaC* genes [20,31]. However, the molecular basis of this synthetic lethal phenotype remains unknown. To address this issue, we isolated extragenic Ts^+^ chromosomal suppressor mutations. Ts^+^ suppressors were obtained at a frequency of 10^−6^ to 10^−7^ and analyzed further after marking with Tn*10*. Three Ts^+^ suppressors were retained. DNA sequence analysis demonstrated that the SR25560 Δ(*lapD waaC*) Ts^+^ suppressor-carrying strain had a single amino acid Leu-to-Pro substitution at amino acid position 651 of LptD (Table 2). Another isogenic Ts^+^ suppressor-carrying SR25561 was found to have a single amino acid substitution, Tyr 243 to His, in the coding sequence of the *nlpI* gene (Table 2). However, the third Ts^+^ suppressor-carrying strain SR25562 identified two Tn*10* linked mutations, LptD L651P and BamC F248L, and was not analyzed further. LptD is an outer membrane protein that assembles LPS in the OM, together with the OM lipoprotein LptE [44]. NlpI is an OM lipoprotein that interacts with the tail-specific periplasmic protease Prc and acts as an adapter to mediate the proteolysis of MepS hydrolase by Prc [45,46,47]. NlpI is suggested to bring endopeptidases close to the complexes involved in PGN synthesis, thereby spatially connecting peptidoglycan hydrolysis to PGN expansion [46,47]. Transduction of the F248L *nlpI* mutation into the wild-type W3110 conferred an osmosensitive phenotype, which could be suppressed by the *nlpI*-expressing plasmid, as is known for the Δ*nlpI* phenotype [48], and hence is a recessive mutation.

**Figure 8:**
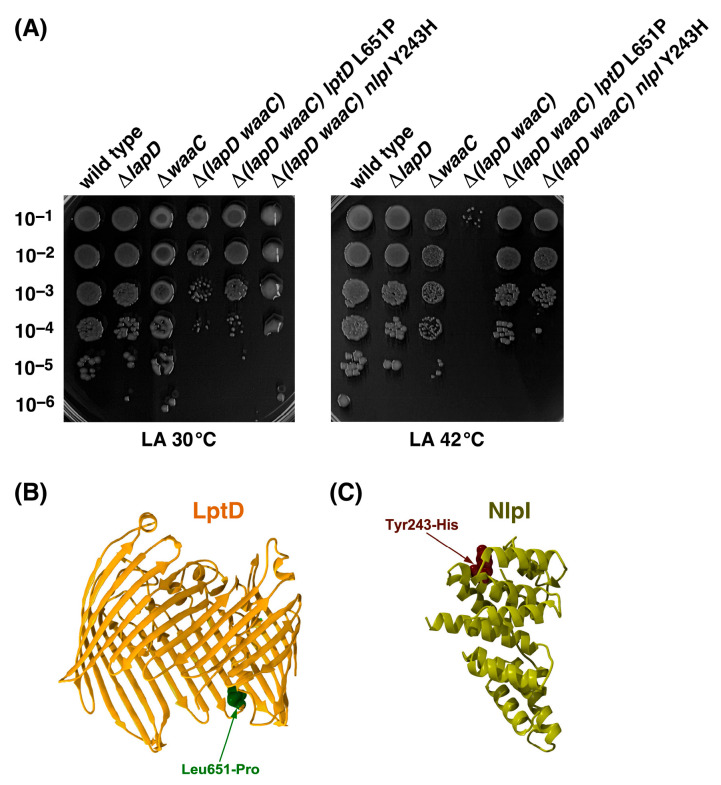
Single amino acid substitutions in either *lptD* or *nlpI* can overcome the synthetic lethality of Δ(*lapD waaC*) bacteria. (**A**) The growth of isogenic cultures of the wild type, its Δ*lapD*, Δ(*lapD waaC*), Δ(*lapD waaC*) *lptD* L615P, and Δ(*lapD waaC*) *nlpI* Y243H derivatives was quantified by spot dilution on LA medium at 30 and 42 °C. The relevant genotypes and incubation temperatures are indicated. A representative image of the spot dilution assay after 24 h of incubation is shown. (**B**) Positions of single amino acid substitutions in the structures of LptD PDB 4RHB and (**C**) NlpI PDB 1XNF are shown.

The restoration of Δ(*lapD waaC*) growth at 42 °C by suppressor mutation mapping to *lptD* and *nlpI* was quantified using spot-dilution assays. Thus, isogenic cultures of wild-type, Δ*lapD*, Δ(*lapD waaC*), and two suppressor-carrying strains, SR25560 and SR25561, were grown under permissive growth conditions at 30 °C in LB medium and examined for growth at 30 °C and 42 °C on LA plates using spot-dilution assays. The results of these experiments revealed more than a 10^4^-fold increase in colony-forming ability at 42 °C in Δ(*lapD waaC*) *lptD* L651P and Δ(*lapD waaC*) *nlpI* Y243H strains compared to the parental Δ(*lapD waaC*) strain (Figure 8). As expected, the Δ(*lapD waaC*) strain was unable to propagate at 42 °C and formed mucoid colonies at 30 °C, similar to the parental Δ*waaC* strain. Interestingly, the Δ(*lapD waaC*) *lptD* L651P strain SR25560 exhibited a more robust (10-fold) suppression at 42 °C than the Δ(*lapD waaC*) *nlpI* Y243H strain SR25561. The LptD L651P suppressor mutation also abolished the mucoid phenotype at 30 °C (Figure 8). The mucoid phenotype is a known phenotype of deep rough mutants lacking heptose I due to the induction of capsular polysaccharide synthesis [49]. Isolation of suppressor mutation mapping to the *nlpI* gene links LapD to PGN biosynthesis, and the suppressor mutation in the *lptD* gene could probably improve LPS translocation to the OM, which we have previously shown to be impaired in Δ*lapD* bacteria [20]. Thus, the function of LapD is not only linked to the regulation of fatty acid biosynthesis but also to ensure coordination with PGN and LPS biosynthesis.

### 2.10. Overexpression of Either the acpP Gene or the accB Gene Overcome Cell Morphology Defects of ΔlapD Bacteria

**Figure 9:**
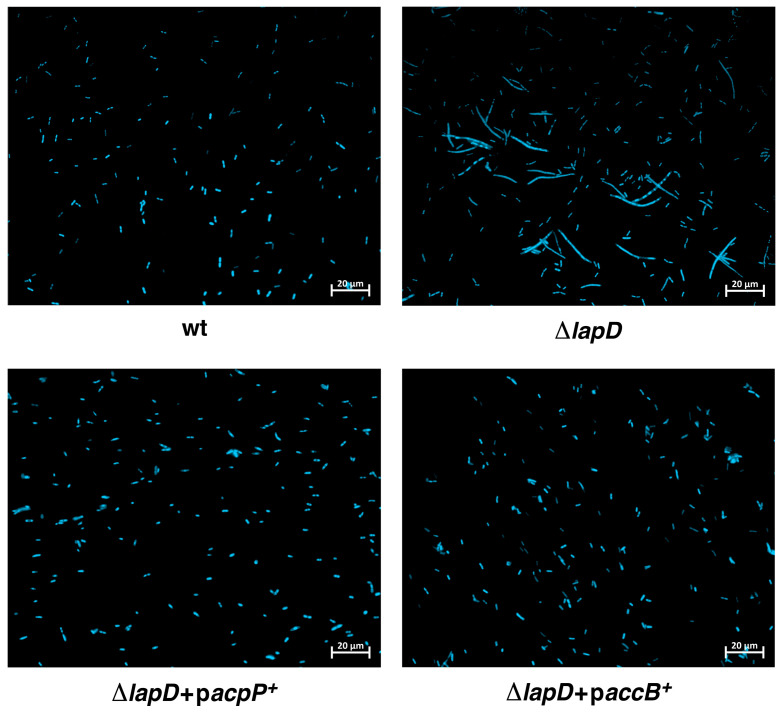
Overexpression of either *acpP* or *accB* genes rescues the cell morphology defects of Δ*lapD* bacteria. Microscopic images of the wild type, its isogenic Δ*lapD* with the empty vector, and Δ*lapD* expressing either *acpP* or *accB* genes. Bacterial cultures were grown overnight at 37 °C, diluted 1:100 into fresh LB, and grown until an OD_600_ of 0.1 at 37 °C. IPTG (75 µM) was added, and the cultures were grown for another 2 h. Cultures were pelleted, and bacterial cells were stained with DAPI and imaged using fluorescence microscopy at ×1000 with a 20 µm scale.

Δ*lapD* bacteria are known to exhibit morphological defects; however, the underlying reasons remain unknown [25]. As cell size is significantly correlated with fatty acid synthesis [23], we rationalized that multicopy suppressors of Δ*lapD* bacteria such as *acpP* and *accB* could act by restoring fatty acid levels. Therefore, we examined their effects on cell morphology. These experiments revealed that Δ*lapD* bacteria in the W3110 background exhibited a filamentous phenotype, which was repressed by overexpression of either *acpP* or *accB* genes (Figure 9). Thus, these experiments allow us to postulate that acyl-ACPs generated by AcpP might be a limiting factor in Δ*lapD* mutants for cellular processes involved in either cell division directly or regulation of fatty acid synthesis, as AcpP interacts with several proteins in these two pathways. The suppression of cell morphology defects by overexpression of the *accB* gene can be explained by disturbing stoichiometry between different subunits of ACC enzyme, which can lead to loss of activity of the enzyme and consequent reduction in fatty acid biosynthesis.

### 2.11. Overexpression of Either acpP or menI Overcomes Vancomycin Sensitivity of ΔlapD Bacteria

**Figure 10:**
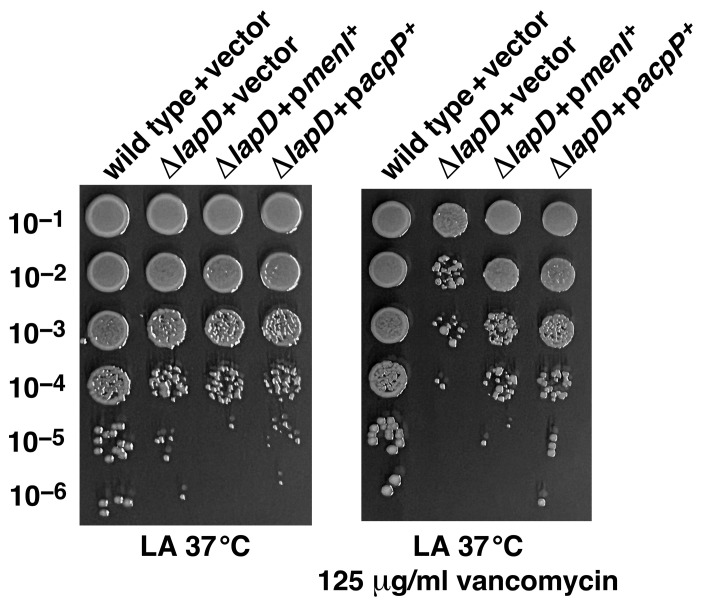
Overexpression of either *menI* or *acpP* genes can suppress vancomycin sensitivity of Δ*lapD* bacteria. The growth of isogenic cultures of wild type with vector alone, its Δ*lapD* derivative with empty vector, and Δ*lapD* bacteria carrying a plasmid expressing either *menI* or *acpP* genes was quantified by spot dilution on LA with and without vancomycin supplementation. The relevant genotypes and vancomycin concentration are indicated.

We previously reported that *acpP* overexpression effectively suppresses the Ts phenotype of Δ*lapD* bacteria. However, Δ*lapD* bacteria also exhibit sensitivity to antibiotics, such as vancomycin [20]. Nevertheless, whether *acpP* overexpression or that of some other gene can restore vancomycin resistance has not been investigated in the W3110 background. Here, we performed a multicopy screen to identify genes whose overexpression confers resistance to vancomycin in Δ*lapD* bacteria. DNA sequence analysis and retransformation identified *acpP* and *menI* genes. This suppression was further quantified by growth analysis using a spot-dilution assay in a growth medium supplemented with 125 µg/mL vancomycin and 75 µM IPTG. The data presented demonstrate that the induction of *menI* expression restores vancomycin resistance in Δ*lapD* bacteria, similar to that observed with *acpP* overexpression (Figure 10). MenI belongs to the 4-hydroxybenzoyl-CoA thioesterase subfamily of the hot-dog fold superfamily of proteins [50,51]. MenI has been reported to exhibit activity toward a wide range of acyl-CoA thioesters, and its role in the menaquinone biosynthetic pathway has been confirmed [51]. However, as MenI overproduction restores vancomycin resistance, it is likely that moderate induction of thioesterase, due to its degenerate activity, could dampen increased fatty acid synthesis in the absence of LapD and hence is relevant to the identification of MenI as a dosage-dependent suppressor of Δ*lapD* bacteria. Furthermore, although acyl carrier protein is a highly abundant protein, it might be limiting in Δ*lapD* bacteria because of its multiple roles in LPS, fatty acid biosynthesis, and potentially cell division.

### 2.12. Overexpression Signal Sequence-Less TesA (TesA’) Overcome Cell Morphology Defects of ΔlapD Bacteria

**Figure 11:**
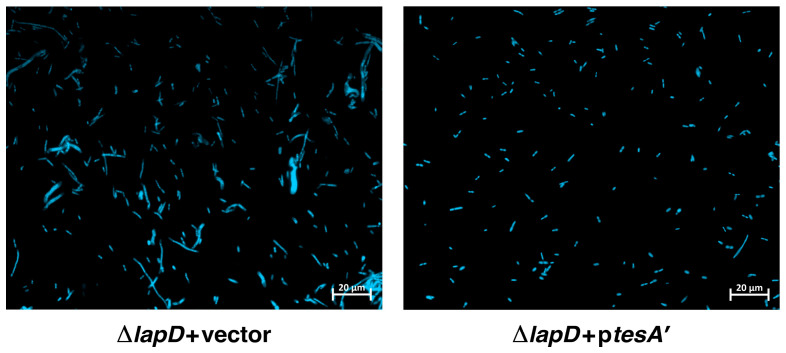
Induction of signal sequence-less TesA′ restores normal cellular morphology of Δ*lapD* bacteria. Microscopic images of the wild type, its isogenic Δ*lapD* with the empty vector, and Δ*lapD* expressing the *tesA*′ were obtained as described in legend to Figure 6. bacterial cells were stained with DAPI and imaged using fluorescence microscopy at ×1000 with a 20 µm scale.

As MenI with degenerate thioesterase activity can suppress some of the defects of Δ*lapD* bacteria, we reasoned that overexpression of a well-characterized thioesterase, such as TesA, would suppress some phenotypic defects of such bacteria; hence, we expressed the *tesA* gene in the cytoplasm. Overexpression of *tesA* has been reported to change the fatty acid composition when expressed in the cytoplasm of *E*. *coli* [52]. As TesA is a periplasmic protein, we cloned the *tesA* gene lacking its signal sequence (*tesA*′) downstream of the tightly regulated p*tac* promoter using the pTTQ18 vector. The *tesA*′-expressing plasmid DNA and the empty vector were used to transform Δ*lapD* to evaluate the impact on cell morphology and *tesA*′ in the presence of 50 µM IPTG as an inducer of gene expression. These experiments revealed that *tesA*′ overexpression suppressed the filamentous morpholgy of Δ*lapD* bacteria (Figure 11).

### 2.13. Induction of Either the Acyl-Carrier Protein or AccB Subunit of the Acetyl-CoA Carboxylase Enzyme or TesA’ Represses Phospholipid Biosynthesis in ΔlapD Bacteria

**Figure 12:**
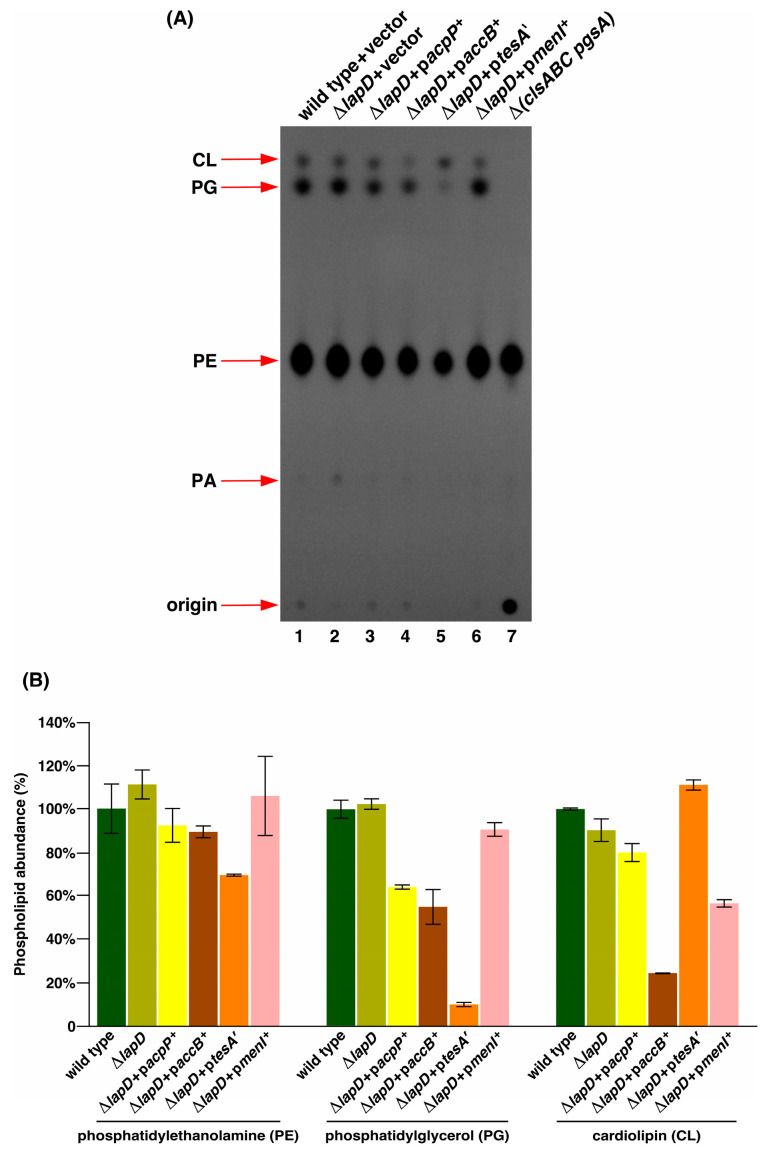
Induction of expression of either *acpP*, *accB* or *tesA*′ genes repress phospholipid biosynthesis in Δ*lapD* bacteria. (**A**) Thin-layer chromatography of ^32^P-labeled PLs extracted from isogenic strains carrying either vector alone or individually expressing *acpP*, *accB*, *tesA*′, and *menI* genes. As a control, ^32^P-labeled PLs were extracted from the RU857 strain, which cannot produce CL and PG and synthesizes only PE, serving as a marker for three major PL species. A representative autoradiogram is presented. The arrows indicate the positions of major PL species. (**B**) Bar graph representing the relative amounts of CL, PG, and PE species quantified using densitometry from three biological replicates. Error bars represent a S.E. of three independent measurements.

As shown in the above sections, overexpression of *acpP*, *accB* and *tesA*′ can restore normal cell morphology and *menI* overexpression can restore vancomycin resistance of Δ*lapD* bacteria, so we further explored their impact on PL amounts by TLC analysis. Densitometric quantification of different PL species revealed that the wild-type strain contained approximately 77.69% ± 3.6 PE, followed by 17.93% ± 0.8 PG and 4.2% ± 0.4 CL (Figure 12 lane 1). These values are consistent with the known abundance of PLs in wild-type *E. coli* K-12 strains [53]. Quantification of different PL species further revealed that overexpression of either *acpP* or *accB* genes reduced the overall amounts of acidic phospholipid species, PG and CL (Figure 12). The impact of *accB* overexpression caused a more significant decrease in CL and PG amounts, with nearly 24% and 54% compared to the wild type, respectively (Figure 12). The induction of *accB* gene expression also led to a decrease in phosphatidylethanolamine (PE) presence. These experiments also revealed a drastic reduction in the PE and PG species of PL when the expression of *tesA*′ was induced (Figure 12). Overexpression of *accB* can disturb the stoichiometric balance of ACC enzyme subunits, leading to the inhibition of ACC enzyme activity [32,33]. However, the induction of *menI* gene expression did not significantly alter PE and PG amounts. The main impact of MenI induction was the reduction in CL species, which was 56% compared to that of the isogenic wild type (Figure 12 Lane 6). These experiments also revealed that Δ*lapD* bacteria were not impaired in the regulation of PL, as no significant differences were observed in the abundance of PE, PG, or CL (Figure 12 Lane 2). However, Δ*lapD* bacteria do accumulate phosphatidic acid (PA) species, although less than 1%, which were not observed in PL extracted from the wild type (Figure 12 Lane 2 vs. Lane 1).

Thus, the reduction in the amount of PL by the overproduction of either AcpP or AccB can adjust the balance between LPS and PL amounts, since Δ*lapD* bacteria have less LPS in the OM, which helps explain the mechanism of suppression and the need to achieve homeostatic control of these essential elements of the cell envelope.

### 2.14. Overexpression of the acpP Gene Suppresses Elevated RpoE-Regulated Envelope Stress Response of ΔlapD Bacteria

**Figure 13:**
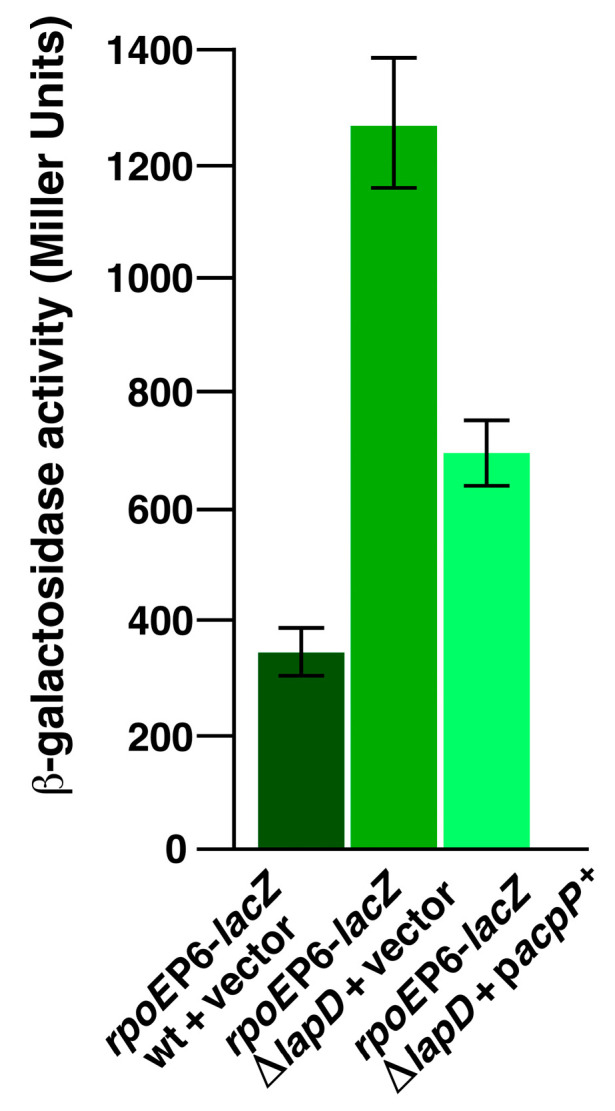
Overexpression of the *acpP* gene represses elevated envelope stress-responsive *rpoE* promoter activity. Exponentially grown wild type, its isogenic Δ*lapD* derivative with the vector alone, and its derivative expressing the *acpP* gene, carrying the single-copy chromosomal *rpoE*P6-*lacZ* fusion, were analyzed for the *β*-galactosidase activity. The cultures were adjusted to an OD_595_ of 0.05. IPTG (75 µM) was added to induce *acpP* expression, and the cultures were allowed to grow in LB medium at 37 °C. Aliquots of samples were drawn after different time intervals and used to measure the *β*-galactosidase activity. Error bars represent a S.E. of three independent measurements.

It is well established that RpoE responds to cell envelope defects in the periplasm, misfolding of OMPs, and defects in LPS and PL regulation [11,15,54,55]. Consequently, deletion of the *lapD* gene, quite like when LPS biosynthesis is dysfunctional in *lapB* or *lapC* mutants, causes elevated RpoE induction [20]. As mild induction of *acpP* gene was found to overcome cell morphology and vancomycin sensitivity defects, we examined whether its overexpression affected RpoE activity. This was measured by estimation of *rpoE*P6-*lacZ* activity in the wild type, Δ*lapD*, and its derivative carrying the inducible *acpP* gene. The *rpoEP6* promoter is well characterized, is positively autoregulated by the RpoE sigma factor [56] and is induced approximately by a 4-fold in Δ*lapD* derivative (Figure 13). Mild induction of *acpP* gene reduced RpoE activity by nearly 50% (Figure 13). These results are consistent with *acpP* acting as a multicopy suppressor of various phenotypic defects, including the reduction in elevated PL amounts and overcoming cell morphology defects in Δ*lapD* bacteria.

### 2.15. Essentiality of the mepS Gene in ΔlapD Bacteria

**Figure 14:**
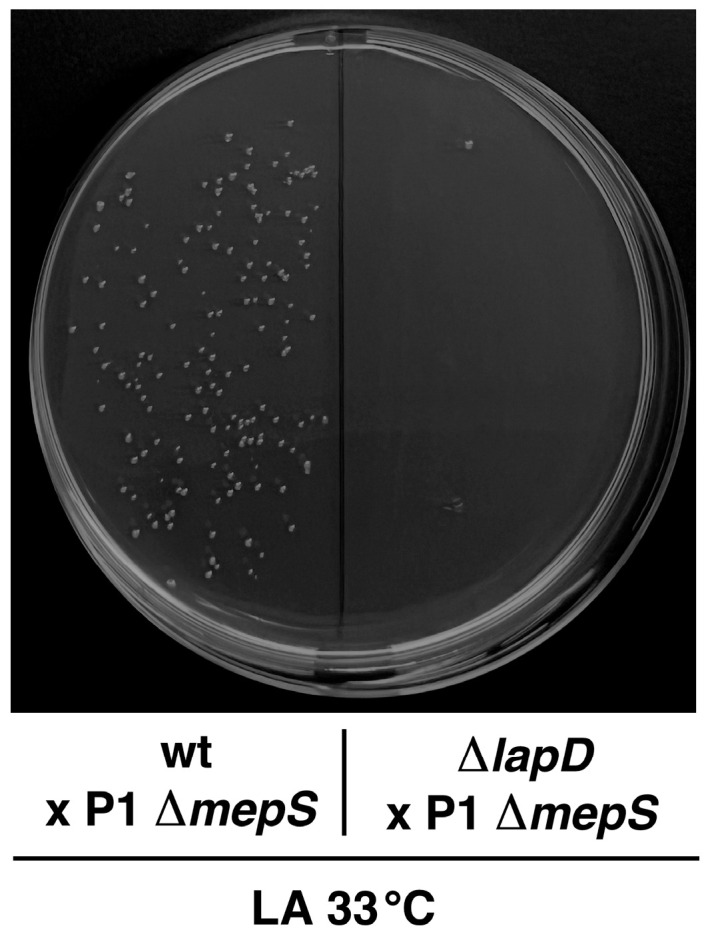
Peptidoglycan hydrolase MepS is essential in the absence of LapD. A representative picture of colonies obtained by bacteriophage P1-mediated transduction, when the Δ*mepS* mutation was introduced into the wild type and its isogenic Δ*lapD* derivative. LA plates were incubated for 24 h at 33 °C.

As suppressor mutation mapping to the *nlpI* gene can overcome the synthetic lethality of Δ(*lapD waaC*) bacteria, we reasoned that it could be at the level of regulation of *mepS*, since NlpI is required for MepS proteolysis by Prc protease. It has been shown that the hydrolytic activity of MepS is associated with murein incorporation during cell growth [46,47]. Thus, we examined whether MepS is required in the absence of LapD. To address this possibility, a Δ*mepS* derivative was constructed in the wild type and used in transduction experiments. Hence, parallel bacteriophage P1-mediated transductions were performed in wild-type W3110 and its Δ*lapD* derivative SR25204. A deletion *mepS* mutation could be readily introduced in the wild-type strain but not in the Δ*lapD* background, allowing us to conclude that MepS is essential in the Δ*lapD* background (Figure 14). Similar synthetic lethality results were obtained when reciprocal transductions were performed by attempting to introduce the Δ*lapD* mutation in a Δ*mepS* background. These results confirm the essential role of murein endopeptidase MepS in the absence of LapD. These findings further support the role of LapD in coordinating LPS, PL, and PGN biosynthesis to maintain the homeostasis of these three essential components of the cell envelope in *E*. *coli*.

## 3. Discussion

The biosynthesis of two essential cell envelope components, LPS and PLs, is coupled because they share a common metabolic precursor and are maintained in a tight balance, with excess of either component leading to lethality [8,57]. However, the mechanisms underlying this phenomenon are not sufficiently clear. In this direction, it was shown that LapD physically interacts with several Fab enzymes, components of the acetyl-CoA carboxylase enzyme, and those involved in phospholipid and LPS biosynthesis [20]. However, the precise function of LapD remains elusive. Furthermore, the molecular basis of the lethality of Δ(*lapD lpxM*) and Δ(*lapD waaC*) mutational combinations, purportedly with involvement in cell division or PGN synthesis, has not been elucidated. Notably, these studies used BW25113 as the parental strain, which has a nonfunctional fatty acid regulator FabR [20,25,26,30,58], which can confound the results. Here, we reconstructed Δ*lapD* derivatives in W3110, which is FabR^+^, and showed that suppressor-free Δ(*lapD lpxM*) derivatives can be constructed in minimal medium at 30 °C, but not in LA medium. This paved the way to address the molecular basis of their lethality on LA medium by isolating single-copy extragenic chromosomal suppressor mutations and analyzing changes in phospholipid and fatty acid composition. Additionally, the Δ(*lapD waaC*) combination in W3110, which exhibited conditional synthetic lethality at temperatures above 42 °C, was used to isolate extragenic suppressors. Furthermore, dosage-dependent suppressors that overcame the cell morphology defects, and vancomycin sensitivity of Δ*lapD* bacteria were characterized.

Mapping of extragenic chromosomal suppressors that bypassed the lethality of Δ(*lapD lpxM*) bacteria in rich medium identified that the majority of suppressor mutations mapped to *accA*, *accC*, and *accD* genes, which encode various subunits of the essential enzyme acetyl-CoA carboxylase transferase. In fatty acid biosynthesis, the first committed step of fatty acid synthesis is the carboxylation of acetyl-CoA by the acetyl-CoA carboxylase enzyme complex to produce malonyl-CoA [28]. This initiation step, catalyzed by the ACC enzyme, is a rate-limiting step that is subjected to growth phase regulation, and its activity is inhibited by acylated derivatives of AcpP [32,33,59]. Loss-of-function mutations in the ACC complex will lead to a reduced rate of fatty acid synthesis and changes in phospholipid amounts. Indeed, complementation of *acc* mutants by single-copy cosmids carrying wild-type genes established that our suppressor mutations were loss-of-function mutations. The identification of suppressor mutations in the ACC complex is not unique to Δ(*lapD lpxM*) but reflects that late acyltransferases required for the completion of lipid A synthesis are critical for balanced LPS and phospholipid biosynthesis. More than three decades ago, work in our group identified the extragenic suppressors of Δ*lpxL* (then *htrB*), which overcame its lethal phenotype at elevated temperatures, to map to the ACC complex [60]. LpxL is required for the incorporation of the lauroyl acyl chain; hence, Δ*lpxL* strains have a majority of tetraacylated species in the lipid A part [31]. These results are also supported by the recent independent isolation of suppressor mutations of *lapD* mutant bacteria mapping to the ACC complex [29].

Analyses of the FFA composition of Δ(*lapD lpxM*) bacteria showed that they accumulated nearly 2-fold excess of unsaturated C16:1 palmitoleic acid compared to the wild type as well as more than 2.5-fold increased amount of myristic acid, which were reversed by suppressor mutations mapping to *accA* and *accC* genes when grown at 30 °C. Furthermore, a single amino acid exchange of AccC Val299 to Ala in Δ(*lapD lpxM*) bacteria resulted in more than a 2-fold increase in C18:1 *cis*-vaccenic acid amounts compared to those in the suppressor-free parental Δ(*lapD lpxM*) derivative. These large-scale changes in fatty acid composition in Δ(*lapD lpxM*) bacteria with suppressor mutations in the ACC complex could help maintain a balance between LPS and PL amounts. It has been shown that acyl-ACP with *cis*-vaccinate can be a potent inhibitor of the ACC complex; hence, feedback inhibition could result in a reduction in the rate of fatty acid balance [33]. This can be a physiologically relevant manner of overcoming synthetic lethality associated with the Δ(*lapD lpxM*) mutational combination. Notably, no major differences in the ratio of either saturated versus unsaturated fatty acids or in terms of the overall pattern of fatty acid composition were discernible between isogenic wild-type and Δ*lapD* bacteria, and the main differences were only observed when comparing Δ(*lapD lpxM*) bacteria with and without suppressor mutations.

Analysis of phospholipid composition revealed the most striking differences in composition and relative abundance, shedding further light on the potential reasons for the lethality of the Δ(*lapD lpxM*) combination. Even under permissive growth conditions, Δ(*lapD lpxM*) bacteria contained more acidic PLs than the parental strains, resulting in nearly a 3-fold increase in CL levels and approximately 60% increase in PG amounts. These results allow us to conclude that the accumulation of increased species of acidic PLs could be one of the major contributors to the lethality associated with the Δ(*lapD lpxM*) mutational combination. In support of this model, suppressor mutations in various subunits of the ACC complex significantly reversed the increase in the acidic phospholipid levels. Although no major differences in the relative abundance of PE, PG, and CL species between the wild type and its isogenic Δ*lapD* derivative were observed, a significant phosphatidic acid (PA) accumulation was found in these bacteria. PA serves as a universal precursor of glycerophospholipids (GLPs) [22], and its accumulation in Δ*lapD* bacteria suggests a requirement for LapD in controlling the amount of this precursor for PL synthesis, probably by controlling the activity of CDP-diglyceride synthetase encoded by the *cdsA* gene. Indeed, mutations in *cdsA* are known to lead to the accumulation of increased amounts of PA [61]. CDP-diacylglycerol is positioned at a branch point in the lipid biosynthetic pathway, where it reacts with *sn*-glycerol 3-phosphate to form phosphatidylglycerophosphate, or with L-serine for the synthesis of phosphatidylserine [62]. It is the source of the phosphatidyl group for all major PLs in *E. coli*.

**Figure 15:**
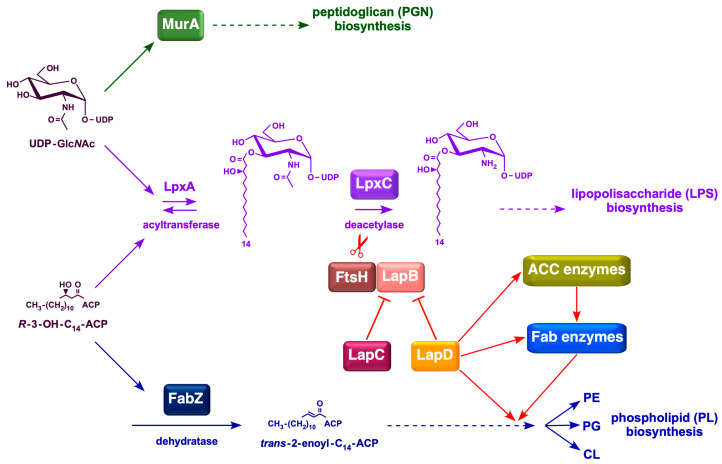
Model of coupled biosynthesis of three essential components of the cell envelope of *E*. *coli*. The shared precursors and initial enzymes of the three biosynthetic pathways are depicted. The importance of LapD at the interface of LPS and phospholipid/fatty acid synthesis is indicated by the arrows.

This study also showed that LapD is involved in coordinating LPS, PL, and PGN synthesis (Figure 15). It is based on the isolation of suppressor mutations of conditional synthetic lethality of Δ(*lapD waaC*) mutants, which allow growth at temperatures above 42 °C. These suppressors were mapped to *lptD*, *nlpI*, and *bamC* genes. LptD is essential for bacterial growth, is a member of the RpoE regulon, and is required for the assembly of LPS in the OM [7,63]. Suppressor mutation in the *lptD* gene could potentiate better assembly of LPS in the OM, as a large fraction of LPS is retained in the IM in Δ*lapD* bacteria, and truncation of LPS due to the concomitant absence of WaaC could further limit its translocation, in accordance with our earlier results [20]. Consistent with the isolation of suppressor mutation in the gene encoding the LPS transporter LptD in the Δ(*lapD waaC*) background, we previously showed that increasing LPS amounts by introducing either *lapB* loss-of-function mutations or stable variants of LpxC can overcome vancomycin sensitivity of Δ*lapD* bacteria [20]. In contrast, NlpI is known to regulate the turnover of PGN hydrolases by the periplasmic protease Prc [45,46,47]. NlpI has been shown to bind to Prc and MepS PGN hydrolase by acting as an adaptor [46,47]. Consistent with these results, we showed that MepS becomes essential in the absence of LapD. These findings are supported by the inability to isolate transposon insertion mutations in *mepS* in the whole-genome saturated mutagenesis of a Δ*lapD* strain [26].

We further speculate that the lethality of the Δ(*lapD lpxM*) combination could also arise from dysfunctional central metabolism and cell division machinery. This notion is supported by the isolation of suppressor mutations mapping to *aceK* and *mukE* genes that allow the growth of Δ(*lapD lpxM*) bacteria. Isocitrate dehydrogenase kinase/phosphatase (AceK) controls the branch point between two pathways of central metabolism, namely the TCA cycle and glyoxylate cycle, by controlling the activity of isocitrate dehydrogenase (ICDH) [64]. Its function is central to the successful adaptation and growth of *E*. *coli* and related genera on acetate and fatty acids. Suppressor mutation in the *aceK* gene is located next to the catalytically essential amino acid residue D475, whose inactivation abolishes the kinase activity of AceK [37]. If the Y474S alteration abolishes AceK kinase activity, it would prevent the inactivation of IDH and hence, its diversion to the glyoxylate cycle. *E*. *coli* can utilize exogenous fatty acids, and growth on fatty acids requires a glyoxylate shunt to permit the entry of the acetyl-CoA products of the β-oxidation into the TCA cycle [65]. Thus, the suppressor mutation in the *aceK* gene reveals a link between LapD and the generation of acetyl-CoA. This, in turn, can affect the ability of Δ(*lapD lpxM*) bacteria to use exogenous fatty acids in the fatty acid degradation pathway to generate acetyl-CoA. Thus, LapD is a multitasking protein involved in the coordination of central metabolism, LPS, PL, and PGN synthesis (Figure 15).

It is well known that *E. coli* size is dependent on fatty acid/PL levels [23,66,67]. Consequently, increased fatty acid/PL availability results in larger cell size and cell expansion. In support of LapD involvement in cell elongation, four major observations were made: first, increased expression of the central regulator of fatty acid/PL synthesis AcpP suppresses cell morphology defects, and the isolation of the suppressor mutation of Δ(*lapD lpxM*) bacteria to the *mukE* gene and co-purification of several Muk proteins with LapD [20]. The second reason for the cell morphology defect could be due to interaction with the essential MukB protein component of the structural maintenance of the chromosome complex, which interacts with the AcpP protein, which is required for its ATPase activity and MukBEF function in vivo [68]. The third reason for the cell morphology defect could be the requirement for MepS in Δ*lapD* bacteria. MepS plays an essential role in cell expansion. As discussed above, MepS, in addition to promoting cell elongation, links LapD to PL, LPS, and PGN homeostasis. Furthermore, it has been shown that elevated MepS activity can interfere with the activation of septal PG biogenesis by the divisome [69]. In support of these observations, MepS levels were shown to be stabilized in Δ*lapD* bacteria [70]. The fourth reason comes from the measurement of PL amounts in Δ*lapD* bacteria when AccB or AcpP are overproduced, that showed a reduction in PL species, which is simultaneously associated with the restoration of normal cell morphology.

Finally, in this study, we addressed the molecular basis of the synthetic lethality of Δ(*lapD lpxM*) and the conditional lethality of the Δ(*lapD waaC*) combination using genetic and biochemical assays. The PL and fatty acid composition suggested that Δ(*lapD lpxM*) lethality could be due to an excess of PL, and this can be overcome by suppressor mutations in *accA*, *accC*, and *accD* genes thereby, bypassing this lethality. In support of these results, we also showed that the inhibition of fatty acid biosynthesis by the FabI inhibitor triclosan restored the growth of Δ(*lapD lpxM*) bacteria. Suppressor analysis of Δ(*lapD waaC*) mapping to the *nlpI* gene, whose product regulates MepS murein hydrolase, further demonstrated that LapD coordinates LPS and PGN synthesis. Consistent with this conclusion, we showed that MepS murein endopeptidase is essential in the absence of LapD. Furthermore, the requirement for hexaacylated lipid A in Δ*lapD* could be critical for its survival, since the absence of LpxM renders it pentaacylated and could impede LPS translocation. Consistent with the function of LpxM, it is required for the viability of CL-deficient strains [71,72]. This is further supported by the significant alteration of CL amounts in Δ(*lapD lpxM*) bacteria and their suppressors. However, further studies are required to directly measure alterations in acyl-ACP pools in Δ(*lapD lpxM*) bacteria and their suppressors and measure the impact of suppressor mutations in *acc* genes on the biochemical activity of the ACC complex.

## 4. Materials and Methods

### 4.1. Bacterial Strains, Plasmids and Media

The bacterial strains and plasmids used in this study are described in Table 4. Luria–Bertani (LB) broth and M9 minimal medium (Difco, Franklin Lakes, NJ, USA) were prepared as described earlier [73]. When required, the media were supplemented with ampicillin (100 µg/mL), kanamycin (50 µg/mL), tetracycline (10 µg/mL), or chloramphenicol (20 µg/mL). When needed, vancomycin (125 µg/mL), cerulenin (25 µg/mL), or triclosan (0.01 and 0.05 µg/mL) were added to the growth medium. To induce the expression of various genes, 50 or 75 µM IPTG was supplemented as per the experimental requirements.

### 4.2. Strain Construction and the Isolation of Extragenic Chromosomal Suppressors

Constructions of non-polar deletion derivatives of *lpxM*, *lapD*, and *waaC* have been previously described [20,31]. In this study, W3110 served as the parental wild-type strain, as it has been widely used as the *E. coli* K-12 wild-type strain in LPS structural analysis. Hence, deletion mutations of *lapD*, *waaC*, and *lpxM* were transduced by bacteriophage P1- or T4-mediated transductions into W3110, as described earlier [79,80]. The non-polar deletion derivative of the *mepS* gene was constructed by replacing its coding sequence by the *aph* cassette from the pKD13 plasmid using the λ recombinase system, as described previously [11,76]. To construct deletion combinations of Δ(*lapD lpxM*), Δ(*lapD waaC*), and Δ(*lapD mepS*), bacteriophage-mediated transductions were performed. In the case of Δ(*lapD lpxM*) construction, thusly obtained transductants were plated on M9 minimal agar plates at 30 °C, which was found to provide permissive conditions. As a control, each recipient in the transduction experiments was transformed with a covering plasmid expressing either *lpxM*, *lapD* or *waaC* genes. As Δ(*lapD lpxM*) transductants were only viable on M9 minimal medium, to identify extragenic chromosomal suppressor mutants, several independently obtained suppressor-free cultures grown under permissive growth conditions were plated on LA plates at 37 or 42 °C. From these Δ(*lapD lpxM*) Ts^+^ isolates, 150 putative suppressor-containing strains were retained. Suppressor mutations were marked with mini-Tn*10* Cm [81], and verified that the Tn*10*-linked suppressor mutation breeds true. A previously described cosmid library [15,78] in a single-copy vector was used to identify cosmid clones that could recombine linked Tn*10* Cm, which were used to group 16 out of 23 suppressors in six complementation groups. To identify the position of the linked transposon, Tn*10* and its flanking regions were subcloned from recombinant cosmid clones, and their DNA was sequenced. The same cosmids sets were used for complementation studies. To identify the mutation in a specific gene, chromosomal DNA of 16 suppressor strains was isolated and used to PCR amplify candidate genes, which were then subjected to DNA sequencing. The chromosomal DNA of the remaining seven Ts^+^ Δ(*lapD lpxM*) strains was used for whole-genome sequencing performed by Novogene Europe, as their suppressor mutation could not be marked by a single Tn*10*. To isolate Ts+ suppressors of Δ(*lapD waaC*) strains, cultures were plated at 42 °C on LA medium. The same approach of mapping the position of suppressor mutation of Δ(*lapD waaC*) was used after marking with Tn*10*. Two of the five Ts^+^ suppressor-carrying strains yielded Tn*10* recombinant cosmids, defining regions covering *lptD* and *nlpI* genes. The third suppressor strain yielded cosmids covering two different genomic regions. To identity the gene with the suppressor mutation, the chromosomal region spanning the candidate genes was PCR-amplified and subjected to DNA sequencing.

### 4.3. The Identification of Multicopy Suppressors, Whose Overexpression Overcomes Vancomycin Sensitivity of ΔlapD Bacteria

The complete genomic library of all predicted ORFs of *E*. *coli* cloned in pCA24N (ASKA collection) [75] was used to transform the Δ*lapD* strain SR25204. As Δ*lapD* bacteria exhibit sensitivity to vancomycin, multicopy suppressors were directly selected for the restoration of growth on LA medium supplemented with 125 µg/mL of vancomycin and 75 µM IPTG to induce the gene expression from the P_T5_-*lac* promoter of the pCA24N vector. Vancomycin-resistant colonies were purified and used to isolate plasmid DNA. DNA from such plasmids was used to retransform the Δ*lapD* strain SR25204 on vancomycin-supplemented plates in the presence of 75 µM IPTG. Plasmids that allowed the growth of Δ*lapD* bacteria on vancomycin were retained, and their DNA was sequenced.

### 4.4. The Construction of Plasmid Expressing Signal Sequence-Less tesA Gene

The *tesA* gene lacking its signal sequence (*tesA*′), with Shine–Dalgarno sequences appended at the 5′ end with oligo nucleotides *tesA*_For: 5′-CATTAGgaattcTAAGGAGGACGTACATGGCGGACACGTTATTGATTCTGGGTG-3′ and *tesA*_Rev: 5′-CATTAGggatccTTATGAGTCATGATTTACTAAAGGC-3′, was PCR amplified. PCR products were digested with EcoRI and BamHI and cloned into the pTTQ18 expression vector [77] using the same restriction sites.

### 4.5. Isolation and Analysis of ^32^P-Labeled Phospholipids Species

Overnight cultures, grown in M9 minimal medium at 30 °C [permissive conditions for Δ(*lapD lpxM*) bacteria], were harvested by centrifugation at 4300× *g* for 10 min. Pellets were resuspended in M9 medium at an OD_595_ of ≈0.05 and allowed to grow up to an OD_595_ of 0.25. This was necessitated by the fact that suppressor-free Δ(*lapD lpxM*) bacteria can be propagated on minimal medium and survive only a few doublings in rich medium at 37 °C. Cultures were centrifuged and washed 3X in LB medium and labeled with 2.5 µCi/mL of ^32^P_i_ in 2.5 mL of LB medium until an OD_595_ of 1.0. These growth conditions were necessary because ^32^Pi cannot be efficiently incorporated into the phosphate-rich M9 minimal medium. When required, during the labeling period, 50 µM IPTG was added to induce the expression from the p*tac*-regulated *tesA*′ gene and 75 µM IPTG was added for the expression of P_T5_-*lac* promoter-inducible *menI*, *acpP*, and *accB* genes. The cultures were harvested by centrifugation, and PLs were extracted using the procedure described by Bligh and Dyer, and Ames [82,83]. PL were collected from the lower organic phase and dried under a stream of nitrogen. Dried samples containing PLs were reconstituted in 50 µL of a 2:1 chloroform/methanol mixture. In all these experiments, before the extraction of PLs, an aliquot of the samples was used to measure the total protein concentration using the Pierce BCA protein assay kit (ThermoScientific Poland, Warsaw, Poland). Equivalent amounts (10,000 cpm/lane) of ^32^P-labeled PLs after adjusting for protein concentration were separated on TLC Silica gel 60 F254, aluminum sheets, 20 cm × 20 cm plates (Merck, Mississauga, ON, Canada). The lipids were separated on plates using a mobile phase of chloroform/ethanol/water/tri-ethylamine 30:35:7:35 (*v*/*v*/*v*/*v*) [84]. The plate was dried and exposed to Kodak BioMax XAR X-ray film or exposed to a PhosphorImager screen to visualize and quantify the labeled PLs using the Amersham Typhoon Biomolecular Imager (Cytiva, Uppsala, Sweden). Densitometry analysis of the autoradiograms was performed using the software from Bio-Rad, Warsaw Poland.

### 4.6. Isolation of Fatty Acids and Their Analysis by Gas Chromatography

Free fatty acids were extracted according to an established procedure [85]. Briefly, isogenic bacterial cultures were grown as described for phospholipid extraction without adding ^32^P_i_. 15 mL cultures were harvested by centrifugation, and the frozen pellets were resuspended in 2.5 mL of H_2_O and vortexed. To this mixture, 5 µL of 10 mg/mL heptadecanoic acid (C17:0) (Merck) dissolved in ethanol was added as an internal standard. Fatty acid methyl esters were obtained by treating the dried extract with 0.5 mL of 1.25 M HCl in methanol and incubated at 50 °C for 15 h. Fatty acids were extracted in 1 mL of hexane and analyzed by gas chromatography using a Shimadzu GC-2010 Pro (Kyoto, Japan) with a 30 m × 0.25 mm capillary column. Using an autosampler, a 1 µL sample was injected using a 1:100 split ratio of the helium carrier gas. The column temperature was maintained at 100 °C with an SPL1 temperature of 250 °C and an SPL1 pressure. For calibration, a commercial fatty acid methyl ester mix C8-C22 (CRM18920 Merck, Warsaw Poland) was used to establish the retention time of various peaks, serving as the reference material, and the data were analyzed using the software provided by Shimadzu (Kyoto, Japan).

### 4.7. Examination of Cellular Morphology

For 4′,6-diamidino-2-phenylindole (DAPI) staining, cultures of the wild-type strain, its isogenic Δ*lapD* transformed with the vector alone, or with the plasmids carrying either *acpP*, *accB*, or *tesA*′, were grown in LB medium at 37 °C in the presence of 0.3% glucose, supplemented with the appropriate antibiotic. Overnight cultures were washed and diluted 1:100, and when the OD_595_ reached 0.1, they were supplemented with 75 µM IPTG to induce gene expression from the P_T5_-*lac* promoter. In the case of *tesA*′, the expression was induced with 50 µM IPTG. Cultures were grown up to an OD_595_ of 0.6 under permissive conditions at 37 °C, and aliquots of cultures were centrifuged at 4300× *g* for 5 min, incubated for 10 min in TBS with 10 µg/mL DAPI solution, and immobilized on agarose pads prior to microscopic observation. The cell morphology was examined using epifluorescence and differential interference contrast (DIC) microscopy. Filters used for DAPI: excitation wavelength 370–410 nm, emission wavelength 430–470 nm. Cells were observed using a Zeiss apotome microscope (Carl Zeiss, Jena, Germany).

### 4.8. Immunoblotting and Measurement of β-Galactosidase Activity

To measure the *β*-galactosidase activity, isogenic cultures of the wild type and its Δ*lapD* derivative with the vector alone or the plasmid expressing the *acpP* gene carrying *rpoE-lacZ* promoter fusions were grown overnight under permissive growth conditions. Cultures were adjusted to an optical density OD_595_ of 0.05 and allowed to grow at 30 °C for another 30 min. IPTG (75 µM) was added to induce the *acpP* gene expression. Aliquots of cultures were taken after different intervals and analyzed for *β*-galactosidase activity as described previously [11]. For immunoblotting, isogenic bacterial cultures of wild type and its Δ*lap*D, Δ(*lapD lpxM*), and its derivatives with extragenic suppressor mutations in *acc* genes and as a control Δ*ftsH sfhC21* were grown in M9 minimal medium at 30 °C to an OD_595_ of 0.5. Equivalent amounts of proteins were applied to a 12% SDS-PAGE and transferred onto a membrane for Western blotting. The blots were probed with polyclonal antibodies against LpxC and revealed using a chemiluminescence kit (Thermo Scientific, Warsaw, Poland) according to the manufacturer’s instructions.

## Figures and Tables

**Table 1:** LapD is essential in the absence of myristoyl acyltransferase LpxM, and exhibits conditional synthetic lethality in the absence of heptosyltransferase I WaaC.

Genotype	Number of Transformants		
	P1 Δ*lapD* LA 30 °C	P1 Δ*lapD* M9 30 °C	P1 Δ*lapD* LA 42 °C
Wt	1020	923	ND ^1^
Δ*lpxM*	8	875	ND
Δ*lpxM* p(*lpxM*^+^)	768	901	ND
Δ*waaC*	676	810	17
Δ*waaC* p(*waaC*^+^)	740	720	830

^1^ ND denotes not determined.

**Table 2:** Suppressors of Δ(*lapD lpxM*) and Δ(*lapD waaC*) map to genes involved in fatty acid biosynthesis, peptidoglycan remodeling, and OM assembly of LPS.

Gene	Function	Mutation Position	Amounts of Isolates
Suppressors of Δ(*lapD lpxM*)			
*accA*	acetyl-CoA carboxyltransferase subunit α	K31E (AAA ⟶ GAA)	2
		R175L (CGT ⟶ CTT)	2
*accC*	biotin carboxylase	K40T (AAA ⟶ ACA)	1
		V42I (GTA ⟶ ATA)	1
		R253H (CGT ⟶ CAT)	1
		V299A (GTT ⟶ GCT)	2
		I309F (ATC ⟶ TTC)	1
*accD*	acetyl-CoA carboxyltransferase subunit β	L39P (CTG ⟶ CCG)	1
		S173P (TCG ⟶ CCG)	1
*opgG*	osmoregulated periplasmic glucans biosynthesis protein G	A100T (GCC ⟶ ACC)	2
*aceK*	isocitrate dehydrogenase kinase	Y474S (TAT ⟶ TCT)	1
*mukE*	chromosome partitioning	A122P (GCA ⟶ CCA)	1
**Suppressors of Δ(*lapD waaC*)**			
*lptD*	lipopolysaccharide assembly	L651P (CTG ⟶ CCG)	1
*nlpI*	lipoprotein	Y243H (TAC ⟶ CAC)	1
*lptD* and *bamC*		*lptD* L651P (CTG ⟶ CCG)	1
	outer membrane protein assembly factor	*bamC* F248L (TTC ⟶ CTC)	

**Table 3:** Measurement of relative fatty acid composition by gas chromatography. Data are presented for three biological replicates for both conditions, and the average with S.E is shown.

M9 30 °C						
	wt	Δ*lapD*	Δ(*lapD lpxM*)	Δ(*lapD lpxM*) *accA* K31E	Δ(*lapD lpxM*) *accC* V299A	Δ(*lapD lpxM*) *accD* L39P
C14:0	1.49 ± 0.13	1.88 ± 0.05	4.07 ± 0.04	3.11 ± 0.07	1.03 ± 0.07	2.62 ± 0.04
C16:0	45.01 ± 1.04	45.35 ± 0.24	41.86 ± 0.07	43.17 ± 0.02	41.35 ± 0.06	45.94 ± 0.04
C18:0	0.72 ± 0.03	0.95 ± 0.10	0.39 ± 0.04	0.58 ± 0.01	2.01 ± 0.10	0.61 ± 0.08
C16:1	15.91 ± 0.40	19.32 ± 0.04	30.13 ± 0.20	30.12 ± 0.06	19.68 ± 0.08	20.99 ± 0.01
cyclopropane	17.93 ± 0.02	14.14 ± 0.07	7.89 ± 0.11	6.25 ± 0.12	5.39 ± 0.15	15.26 ± 0.11
C18:1	18.94 ± 1.66	18.36 ± 0.19	15.66 ± 0.06	16.78 ± 0.23	30.54 ± 0.16	14.58 ± 0.20
**LB 37 °C**						
C14:0	3.21 ± 0.03	4.03 ± 0.04	3.85 ± 0.10	3.43 ± 0.03	2.14 ± 0.01	3.71 ± 0.11
C16:0	42.60 ± 1.11	45.17 ± 0.14	43.40 ± 0.16	45.29 ± 0.07	44.58 ± 0.21	44.11 ± 1.94
C18:0	3.21 ± 0.09	3.31 ± 0.08	0.87 ± 0.20	0.77 ± 0.06	1.35 ± 0.04	0.74 ± 0.05
C16:1	31.41 ± 0.73	31.51 ± 0.02	28.78 ± 0.05	31.01 ± 0.14	25.09 ± 0.17	31.63 ± 1.86
cyclopropane	2.18 ± 0.35	2.53 ± 0.08	4.79 ± 0.06	2.85 ± 0.01	2.00 ± 0.04	2.37 ± 0.21
C18:1	17.37 ± 0.629	13.45 ± 0.05	18.31 ± 0.03	16.66 ± 0.17	24.83 ± 0.08	17.43 ± 0.75

**Table 4:** Bacterial strains and plasmids used in this study.

Strains	Genotype	Reference
W3110	λ^−^, *IN* (*rrnD-rrnE*)*1*, *rph-1*	CGSC, Yale
GK1275	W3110 *lpxM*<>*aph*	[31]
SR7336	GK1275 *lpxM*<>*frt*	This study
SR8035	W3110 *waaC*<>*aph*	[31]
SR25196	W3110 *lapD*<>*aph*	This study
SR25204	SR25196 *lapD*<>*frt*	This study
GK6173	SR25204 *lapD*<>*frt lpxM*<>*aph*	This study
SR25236	SR7336 *lpxM*<>*frt lapD*<>*aph*	This study
SR25217	SR25204 *lapD*<>*frt lpxM*<>*aph accD* L39P	This study
SR25218	SR25204 *lapD*<>*frt lpxM*<>*aph accA* K31E	This study
SR25219	SR25204 *lapD*<>*frt lpxM*<>*aph accC* V299A	This study
SR25220	SR25204 *lapD*<>*frt lpxM*<>*aph accD* S173P	This study
SR25224	SR25204 *lapD*<>*frt lpxM*<>*aph accA* R175L	This study
SR25225	SR25204 *lapD*<>*frt lpxM*<>*aph accC* R253H	This study
SR25235	SR25204 *lapD*<>*frt lpxM*<>*aph accA* K31E	This study
SR25240	SR25204 *lapD*<>*frt lpxM*<>*aph accC* V299A	This study
SR25241	SR7336 *lpxM*<>*frt lapD*<>*aph accC* K40T	This study
SR25307	SR25204 *lapD*<>*frt lpxM*<>*aph accA* R175L	This study
SR25438	SR7336 *lpxM*<>*frt lapD*<>*aph accC* I309F	This study
SR25437	SR7336 *lpxM*<>*frt lapD*<>*aph opgG* A100T	This study
SR25443	SR7336 *lpxM*<>*frt lapD*<>*aph opgG* A100T	This study
SR25454	SR7336 *lpxM*<>*frt lapD*<>*aph accC* V42I	This study
SR25452	SR7336 *lpxM*<>*frt lapD*<>*aph aceK* Y474S	This study
SR25458	SR7336 *lpxM*<>*frt lapD*<>*aph mukE* A122P	This study
SR25651	SR25204 *lapD*<>*frt waaC*<>*aph*	This study
SR25660	SR25204 *lapD*<>*frt waaC*<>*aph lptD* L651P	This study
SR25661	SR25204 *lapD*<>*frt waaC*<>*aph nlpI* Y243H	This study
SR25662	SR25204 *lapD*<>*frt waaC*<>*aph lptD* L651P *bamC* F248L	This study
RU857	Δ*pgsA* Δ(*clsABC*) *lpp*2	[74]
SR25199	SR25204 *lapD*<>*frt* + p*acpP*^+^	This study
SR25203	SR25204 *lapD*<>*frt* + p*accB*^+^	This study
SR25870	SR25204 *lapD*<>*frt* + p*tesA′*	This study
SR25872	SR25204 *lapD*<>*frt* + p*menI*	This study
SR25467	W3110 *accA* K31E Tn*10*	This study
SR25468	W3110 *accC* V299A Tn*10*	This study
SR25874	W3110 + pCA24N	This study
SR23799	SR25204 *lapD*<>*frt* + pCA24N	This study
SR25878	W3110 + pTTQ18	This study
SR25880	SR25204 *lapD*<>*frt* + pTTQ18	This study
**Plasmids**	**Genotype**	**Reference**
pCA24N	IPTG-inducible expression vector cm^R^	[75]
pKD3	*oriR6K_g_*, *bla*(Amp^R^), *kan*, *rgnB*(Ter), *cat*	[76]
pKD13	*oriR6K_g_*, *bla*(Amp^R^), *kan*, *rgnB*(Ter)	[76]
pKD46	*araBp*-*gam*-*bet*-*exo*, *bla*(Amp^R^), *repA101*(ts)	[76]
pCP20	ts replicon with inducible FLP recombinase	[76]
pTTQ18	expression vector with LacI represor	[77]
pSR25845	*tesA*′ in pTTQ18	This study
JW1080	*acpP*^+^ in pCA24N cm^R^	[75]
JW3223	*accB*^+^ in pCA24N cm^R^	[75]
JW1676	*menI*^+^ in pCA24N cm^R^	[75]
JW5539	*lapD*^+^ in pCA24N cm^R^	[75]
pREG	cosmid cloning vector	[78]
pSR25460	*accA*^+^ in pREG amp^R^	This study
pSR25461	*accC*^+^ in pREG amp^R^	This study
pSR25469	*accD*^+^ in pREG amp^R^	This study

## Data Availability

The original contributions presented in this study are included in the article. Further inquiries can be directed to the corresponding authors.
